# Incidence of new osteoporotic adjacent vertebral body fractures. A comparison between conservative treatment and vertebral body augmentation (vertebroplasty, kyphoplasty): a systematic review and meta-analysis

**DOI:** 10.3389/fsurg.2025.1594217

**Published:** 2025-05-20

**Authors:** Panagiotis Korovessis, Vasileios Syrimpeis, Alkis Korovesis, Georgios Dimakopoulos

**Affiliations:** ^1^ Spine and Orthopaedics Department, Olympion General Hospital, Patras, Greece; ^2^ Embedded Systems Design and Applications (ESDA) Laboratory, Department of Informatics and Telecommunications, University of the Peloponnese, Patras, Greece; ^3^ University of Patras, Patras, Greece; ^4^ Biostats, Epirus Science and Technology Park Campus, University of Ioannina, Ioannina, Greece

**Keywords:** adjacent fracture, kyphoplasty, vertebroplasty, adjacent, vertebra fracture

## Abstract

**Study design:**

A Systematic Review and Meta-Analysis

**Purpose:**

To compare the incidence of New Adjacent Vertebral Fractures (ANVFs) in elderly patients with Osteoporotic Vertebral Compression Fractures (OVCFs) undergoing either percutaneous vertebral augmentation—via Vertebroplasty (PVP) or Kyphoplasty (PKP)—or Conservative Treatment (CT). Additionally, this study aims to identify potential risk factors associated with ANVFs.

**Hypothesis:**

The incidence of ANVFs does not significantly differ between patients managed with CT and those treated with PVP or PKP.

**Background:**

While the optimal treatment for OVCFs remains debated, PVP and PKP offer immediate stabilization, pain relief, and may help correct vertebral body wedging with minimal complications. However, a review of the literature reveals a limited number of meta-analyses comparing CT with PVP/PKP regarding the incidence of ANVFs.

**Materials and methods:**

Following PRISMA guidelines, a systematic search was conducted across PubMed, Cochrane, Web of Science, Scopus and Science Direct to identify studies published between 2005 and 2024 comparing surgical treatment with CT for ANVFs incidence. Nine studies (five RCTs and four retrospective comparative case-control studies) involving 1,930 patients were included in the analysis.

**Results:**

In RCTs, the analysis indicated a significant difference (*P* < 0.05) in ANVFs incidence favoring the surgical group, with a Relative Risk (RR) of 0.66 (95% CI: 0.44–0.99; *P* = 0.05); in retrospective studies, no statistically significant difference was found between the surgical and CT groups (OR = 0.87, 95% CI: 0.58–1.31; *P* = 0.51). Differences in study parameters such as age, total number of participants, surgical approach (unilateral vs. bilateral), etc. were observed but they could not be accurately tested due to the limited number of studies.

**Conclusion:**

This meta-analysis, for the selected RCTs, shows that vertebral augmentation is associated with a lower incidence of ANVFs compared to CT. On the other hand, in the retrospective studies group there was no significant difference in the incidence of ANVFs between the two treatment groups (CT vs. PKP/PVP). Variations in study parameters, such as patient demographics and surgical techniques, may have affected these results. Further high-quality studies are needed to better understand the long-term effects of different treatment strategies on the incidence of ANVFs.

**Systematic Review Registration:**

PROSPERO (CRD420250509815).

## Introduction

The ideal treatment approach for Osteoporotic Vertebral Compression Fractures (OVCFs) remains a topic of debate. Conservative treatment (CT) is considered the “gold standard” for OVCFs and typically includes rest, analgesics, braces, etc. While CT can help alleviate pain, they may increase the risk of chronic OVCFs (1, 2). The effectiveness of long-term medication is often restricted due to its adverse side effects (gastrointestinal bleeding, hypostatic pneumonia, deep vein thrombosis, etc. (3–11). These concerns are among the reasons why many authors recommend early surgical intervention for OVCFs in the elderly (6, 12). Percutaneous vertebral body augmentation techniques such as percutaneous vertebroplasty (PVP) and percutaneous balloon kyphoplasty (PKP) for treating symptomatic OVCFs are used to stabilize the fractured vertebra and provide pain relief (6, 13).

However, PVP and PKP may result in surgical complications, primarily related to the injection of PMMA including cement leakage and associated neurological injuries on the nerve roots or spinal cord, etc. (14–16).

With the growing use of vertebral augmentation surgical techniques for OVCFs, spine surgeons have raised increasing concerns about the generation of new OVCFs either adjacent to the augmented vertebra (ANVFs), or re-fractures, e.g., fractures at the vertebral augmentation index level (17) or remote new vertebral fractures. Such occurrences often necessitate a new treatment, increasing patient discomfort and imposing a financial burden on families (16–20) ANVFs have a reported risk of 2%–23% in PKP and up to 52% in PVP (21). Several hypotheses have been suggested to explain the rising incidence of ANVFs following vertebral augmentation such as osteoporosis, biomechanical and balance factors, etc.) (22). Some authors suggest that restoring sagittal balance and physiological loading through vertebral augmentation may help reduce ANVFs, which are primarily attributed to underlying osteoporosis and mechanical alterations caused by spinal deformity (16, 20, 23–30). Clinical prediction models have assessed the likelihood of ANVFs following PVP providing risk factors such as a prior history of OVCFs, bone cement leakage to adjacent intervertebral disc, multi-level vertebral augmentation, distribution of PMMA, BMD, BMI, etc. (31). The long-term impact of vertebral augmentation on ANVFs remains a topic of debate with most of the studies showing no statistically significant difference between CT and PVP (32).

Whether OVCFs should be treated surgically or conservatively is controversial (33, 34). Meta-analyses comparing CT vs. PVP/PKP reported primarily on immediate and intermediate pain reduction and functional outcomes and did not address or analyze the occurrence of ANVFs (35–41). Contradictory results have been generated in studies that compared a CT with a PVP with the passage of time (42–44).

Due to the limited literature comparing CT with PVP/PKP in the development of ANVFs and due to the controversies in the related studies, this meta-analysis aims to determine whether the incidence rate of ANVFs after CT is lower than that following PVP/PKP.

## Materials and methods

### Literature search

Following PRISMA (Preferred Reporting Items for Systematic Reviews and Meta-Analyses) guidelines (45), a systematic search was conducted across PubMed, Web of Science, Cochrane, ScienceDirect and Scopus to identify studies published between 2005 and 2024. The process was designed to identify all eligible studies using the following query:

(“vertebroplasty” OR “kyphoplasty” OR “conservative treatment”) AND “new osteoporotic vertebral fractures”

No language filters were used during the search process.

The articles included in this review met the following criteria: Study Design [Randomized Controlled Trials (RCTs) or retrospective cohort studies with a matched control group]; Comparative Analysis [Studies comparing percutaneous vertebroplasty (PVP) or balloon kyphoplasty (PKP) with conservative treatment (CT)]; Patient Population (Studies involving patients with OVCFs); Studies with primary endpoint the incidence of new adjacent OVCFs. In cases, where duplicate or overlapping data were identified in multiple studies, only the study with the most complete and comprehensive data was included in the review.

The criteria for including studies in this review were based on the PICO (46) (Population, Intervention, Comparison, and Outcome) framework. Population (P): Adults or elderly patients with osteoporotic vertebral compression fractures (OVCFs) in the thoracolumbar spine, not caused by neoplasm, trauma, or any other specific condition. Interventional treatment (I): Percutaneous Vertebroplasty (PVP) and Percutaneous Balloon Kyphoplasty (PKP). Comparison-Controls (C): Patients who received Conservative Treatment (CT). Outcome (O): The incidence of adjacent new vertebral fractures (ANVFs) following CT or PVP/PKP.

The inclusion criteria for the Meta-Analysis were as follows: Condition: Symptomatic osteoporotic vertebral compression fractures (OVCFs) in the thoracolumbar spine (T1 to L5 vertebrae); Clinical Presentation: Presence of pain or localized pressure correlating with imaging findings; Diagnostic Confirmation: Preoperative spinal radiographs and MRI confirming new fractures without neurological deficits; Study Size: Studies with more than 30 cases each; Outcome Reporting: Series specifically reporting on new fractures following PVP or PKP and Language: Articles published in English.

The exclusion criteria for the Meta-Analysis were as follows: Study Type: Narrative reviews, systematic reviews, meta-analyses, and case reports; Sample Size: Studies with fewer than 30 participants; Language: Articles published in languages other than English; Intervention: Studies involving additional use of instrumentation; Study Population: Cadaveric studies and studies on pathological fractures, including those caused by hemangiomas, known hematological diseases, infections, or metastatic disease.

From each eligible article, the following information was extracted and documented in an Excel sheet: (1) Authors' name; (2) Year of publication; (3) Type of study; (4) Demographic and baseline characteristics of participants; (5) Type of conservative treatment; (6) Type of surgical treatment; (7) Follow-up details; (8) Outcome data (new OVCF) and (9) Reported complications.

### Selection of studies

The search process was blinded, with only article titles and abstracts reviewed initially. Two independent observers assessed the quality and bias of the retrieved studies independently. Articles were selected based on the inclusion criteria to minimize bias in both study selection and data extraction.

### Key steps in the review process

Initial Selection: Articles were evaluated based on titles and abstracts. Full texts were retrieved for studies with unclear inclusion/exclusion criteria; Resolution of Doubts: If uncertainties persisted, the decision was made through discussion, with a third reviewer brought in if necessary; Scoring: Each primary study was assigned a score independently by the two reviewers. The final score for each study was the average of the two scores.

### Protocol and data extraction

A written study protocol was developed before starting the literature review, including clearly defined eligibility criteria; Two investigators independently extracted relevant data from each trial using a standardized form; The same observers independently extracted data from each article to ensure consistency; This rigorous approach ensured a systematic and unbiased review process. The common characteristics to provide an overview of the 9 studies finally included in the analysis are shown in Tables 1A,B.

**Table 1:** Common clinical characteristics of the 9 included studies in the meta-analysis.

References	Country	Year of publication	Study design	Compared groups CT vs. surgical (PVP/PKP)	PVP/PKP group average age (years)	CT Group average age (years)	PVP/PKP group patients’ number	CT Group patients’ number	Total number of primary OVCFs	Number of augmented vertebrae (PVP/PKP)	PMMA amount per vertebra	Follow-up period for new fracture development
A												
Majid Reza Farrokhi (61)	Iran	2011	RCT	PVP vs. CT	72 (59–90)	74 (55–87)	40	42	190	100	5 ml (range: 1–9 ml)	36 months
S-W Baek (62)	Korea	2015	Retrospective	PVP vs. CT	75.7 ± 6.2	76.5 ± 7.5	91	134	225	112	Not reported	24 months
Wencheng Yang (108)	PR China	2019	Retrospective	PVP vs. CT	64.2 ± 12.2	64.19 ± 12.2	290	270	560	290	3–5 ml	24 months (range: 24–78 months)
Er-Zhu Yang (39)	PR China, USA	2015	RCT	PVP vs. CT	77.1 ± 6.0	76.2 ± 5.6	56	51	123	65	Mean: 4.5 ± 1.2 ml (range: 3–6.5 ml)	12 months
Henrik Teuber (109)	Switzerland	2018	Retrospective	PKP vs. CT	73.5 ± 7	73 ± 10	49	49	98	49	Not reported	12 months
C. A. H. Klazen (36)	Netherlands, Belgium	2010	RCT	PVP vs. CT	75.2 ± 9.8	75.4 ± 8.4	101	101	265	134	4.10 ml (range: 1–9 ml)	4.6 ± 5.4 months (PVP), 6.1 ± 5.9 months (CT)
Xiaodong Yi (110)	PR China	2014	RCT	PVP/PKP vs. CT	70.9 ± 10.04	63.9 ± 15.51	169	121	363	217 vertebrae in 169 patients (90 PVP, 79 PKP)	Not reported	8.95 ± 7.34 months (adjacent), 10.75 ± 8.68 months (non-adjacent)
Chengyue Ji (111)	PR China	2021	Retrospective	PVP/PKP vs. CT	70.26 (56–97)		141	176	384	141 fractures in 141 patients	Not reported	32.46 ± 3.86 months
Rikke Rousing (64)	Denmark	2009	RCT	PVP vs. CT	80 (65–96)	80 (71–93)	25	24	63	PVP group: 31 fractures, CT group: 32 fractures	Not reported	3-month follow-up

**Table:** 

References	Authors institution (Single/Multicenter)	CT group conservative treatment description	Intradiscal cement leakage in adjacent disc (per augmented vertebra)	Unilateral or bilateral approach for PVP/PKP	New adjacent vertebral fractures (ANVFs)	Reported risk factors for new fractures
B						
Majid Reza Farrokhi (61)	Single	Acetaminophen with Codeine, ibuprofen, calcium vitamin D, alendronate and calcitonin	5 (5%)	Both unilateral parapedicular in 35 patients (87.5%) and bilateral transpedicular in 5 patients (12.5%)	CT group (13.3%) was higher than in the PVP group (2.2%; *P* < 0.01)	Lower percentage of new fractures than previous studies, that may be explained by the use of the unilateral approach and the existence of the vacuum phenomenon in some patients, which both require a low volume of cement injection
S-W Baek (62)	Single	Bed rest, analgesics braces, and physical therapy	3 (30%)	Unilateral	CT group: 15/134 patients (11.1%), PVP group: 12/91 patients (13.1%)	The most important factors for new VFs after the initial OVCF are the degree of osteoporosis and altered biomechanics (spinopelvic imbalance) in the fractured area of the spine
Wencheng Yang (108)	Single	Oral analgesics bed rest, physiotherapy, and thoracolumbar brace. Patients in both treatment groups received bisphosphonates, calcium supplementation, and vitamin D	Not reported	Unilateral	42 ANVFs in 37 (13%) of 290 PVP patients, 33 ANVFs in 30 (11%) of 270 CT patients	PVP did not increase the incidence of new VCFs, especially those adjacent to the treated vertebrae, following augmentation with PVP compared with CT. The most important risk factor for NVCFs was osteoporosis and the development of NVCFs was a natural process associated with osteoporosis.
Er-Zhu Yang (39)	Multicenter	Bed rest for the initial 2 weeks then stand up and walk with brace and assistance. For pain medication, nonsteroidal anti-inflammatory drugs (NSAIDs). Additional analgesics: tramadol and morphine would be added in case NSAIDs were not effective	22 (33.8%)	Unilateral	PVP group: 14 (5%) vs. CT group: 12 (4.4%)	In elderly patients with acute OVCF and severe pain, early PVP can offer faster, greater pain relief, and improved functional outcomes for 1 year, with fewer complications compared to conservative treatments.
Henrik Teuber (109)	Single	Not mentioned	Not reported	Bilateral	PKP group: 10 (20.4%) within 1 year, similar to CT group (18.4%)	PKP did not show an increased rate of additional symptomatic adjacent-level VCFs 1 year after surgery when compared to a non-operative control group matched for age, gender, fracture level and bone mineral density. The time for a new adjacent fracture after the index fracture was significantly shorter in the PKP vs. the non-operative group.
C. A. H. Klazen (36)	Multicenter	Analgesics optimized in classification and dose by an internist on a daily basis. Patients in both treatment groups received bisphosphonates, calcium supplementation, and vitamin D	97 (72%)	Bilateral	7 (7.6%) in 91 PVP patients; 11 (12.9%) in 85 CT patients	The incidence of new OVCFs was not different after PVP compared to CT after a mean of 11.4 months follow-up. The only risk factor for new VCFs was the number of VCFs at baseline.
Xiaodong Yi (110)	Single	Pain medication, bed rest, a solf bivalved body brace, and physiotherapy	2 (0.9%)	Bilateral	42 ANVFs (14 PVP/PKP patients) (8.28%), 17 CT patients (14%) (NS)	The incidence of ANVFs was substantially higher but no sooner than these at distant levels in PVP/PKP group. No major risk factors involving new OVCFs have been found.
Chengyue Ji (111)	Single	Not mentioned	Not reported	Both unipedicular and bipedicular approaches	7 (7.6%) new fractures were observed in 91 patients in PVP group. 11 (12.9%) new vertebral fractures in 85 patients treated with CT therapy	The findings suggested that a full use of CT scans, and Hu value of L1 < 50 was independently associated with increased risk of new OVCFs. Moreover, patients with non-spinal fracture history represent an important population to target for secondary fracture prevention.
Rikke Rousing (64)	Single	Physiotherapy if necessary, until discharge, brace treatment if needed	Not reported	Bilateral	2 ANVFs (7.7%) in 26 PVP patients, 1 remote fracture in 24 CT patients (no adjacent fractures)	The majority of patients with acute or subacute painful OVCFs will recover after a few months of CT. The risk of adjacent fractures needs further research.

The protocol for this meta-analysis has been registered in the PROSPERO database (Registration No: CRD420250509815).

The data supporting this meta-analysis have been deposited in the Mendeley Data repository and can be accessed at DOI: 10.17632/t9c5v4859k.1.

### Common clinical characteristics of the selected studies

The primary outcome was adjacent new OVCFs. Perioperative outcomes (PMMA amount injected in each vertebra/per patient); Radiographic outcomes included surgical complications (adjacent intradiscal cement leakage, adjacent new vertebral fractures).

### Statistical analysis

The meta-analysis was conducted in accordance with the recommendations of the Cochrane Collaboration and the guidelines for Quality of Reporting of Meta-analyses (46, 55–57). The analysis of the outcomes was divided to subgroups according to surgical (PVP, PKP) or conservative treatment (CT). RCTs quality was justified using Cochrane Collaboration's “Risk of Bias” tool (58), (Table 2) while for the retrospective studies quality was justified using the Newcastle‒Ottawa scale (NOS) (59) (Table 3). NOS include three areas, patient representation, exposure and outcome determination, and follow-up adequacy, with a maximum total score of 9 for each study. NOS scores of 0–5, 6–7, and 8–9 indicate low, moderate, and high quality, respectively.

**Table 2:** Cochrane scores for the 5 RCTs included in the meta-analysis.

Authors	Random sequence generation	Allocation concealment	Blinding of participants and personnel	Blinding of outcome assessment	Incomplete outcome data	Selective reporting	Other sources of bias
Majid Reza Farrokhi (61)	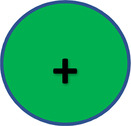	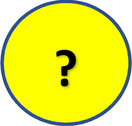	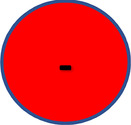	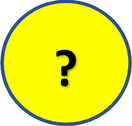	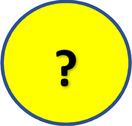	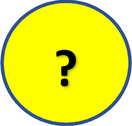	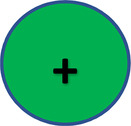
C. A. H. Klazen (36)	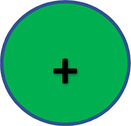	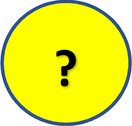	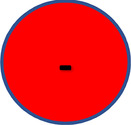	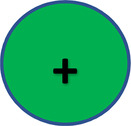	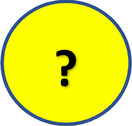	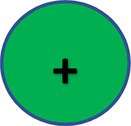	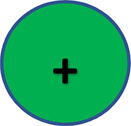
Xiaodong Yi (110)	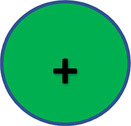	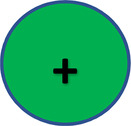	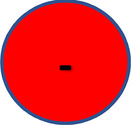	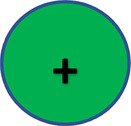	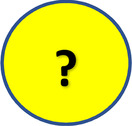	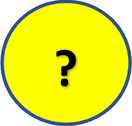	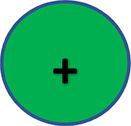
Er-Zhu Yang (39)	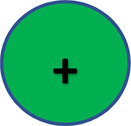	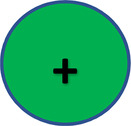	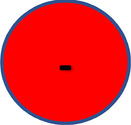	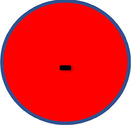	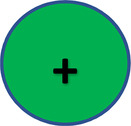	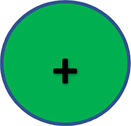	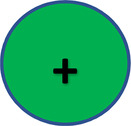
Rikke Rousing (64)	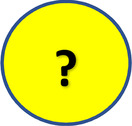	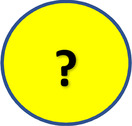	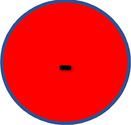	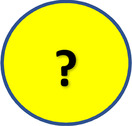	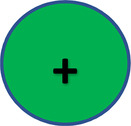	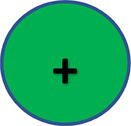	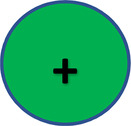

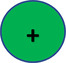
, low risk of bias; 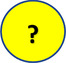, unclear risk of bias; 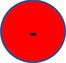, high risk of bias.

**Table 3:** Newcastle Ottawa scores for the 4 retrospective studies included in the meta-analysis.

Author	Year publication	Selection	Comparability	Outcome	Total
Henrik Teuber (109)	2018	4	2	3	9
S-W Baek (62)	2015	4	1	2	7
Wencheng Yang (108)	2019	4	1	2	7
Chengyue Ji (111)	2021	3	2	3	8
Average		3,75	1,5	2,5	7,75

Heterogeneity across the studies was assessed using the chi-square test and *I*^2^ statistic, with *I*^2^ values ranging from 0% to 100% indicating low, moderate, and high heterogeneity at values of 25%, 50%, and 75%, respectively. In this meta-analysis, the pooled estimate was derived under a random-effects model using the inverse variance method (60).

### Risk of bias across the studies

The possibility of publication bias was assessed by analyzing a funnel plot using RevMan 5.4. Symmetry in the funnel plot suggests the absence of publication bias, whereas asymmetry may indicate the non-publication of small trials with negative results or a preference for publishing studies with favorable outcomes.

### Meta-analysis

A meta-analysis of the mean rate of ANVFs between experimental (surgical interventions, PVP/PKP) and controls (CT) was conducted for the nine studies. The analysis was carried out for retrospective design studies and RCTs and aimed to estimate the pooled effect size. We extracted the odds ratios (ORs) to describe the outcomes of interest data, with its 95% confidence intervals (CIs). The pooled OR was estimated for retrospective studies and the relative risk (RR) for RCTs. The conceptual background of the included studies indicated that all estimates should be based on random-effects models and the inverse variance method. A sensitivity analysis was conducted to assess the impact of individual studies on the overall inference. Additionally, an analysis was performed to evaluate the selection of subgroup approaches for vertebral augmentation, such as comparing unilateral and bilateral approaches in relation to the generation of new fractures. The analysis was conducted with the use of RevMan 5.4 and Meta essentials v.1.5, and significance was set at 0.05 in all cases. Heterogeneity was assessed using the *I*^2^ index. Publication bias was assessed using funnel plots and the Egger's test.

## Results

### Literature search and selection of studies

**Figure 1:**
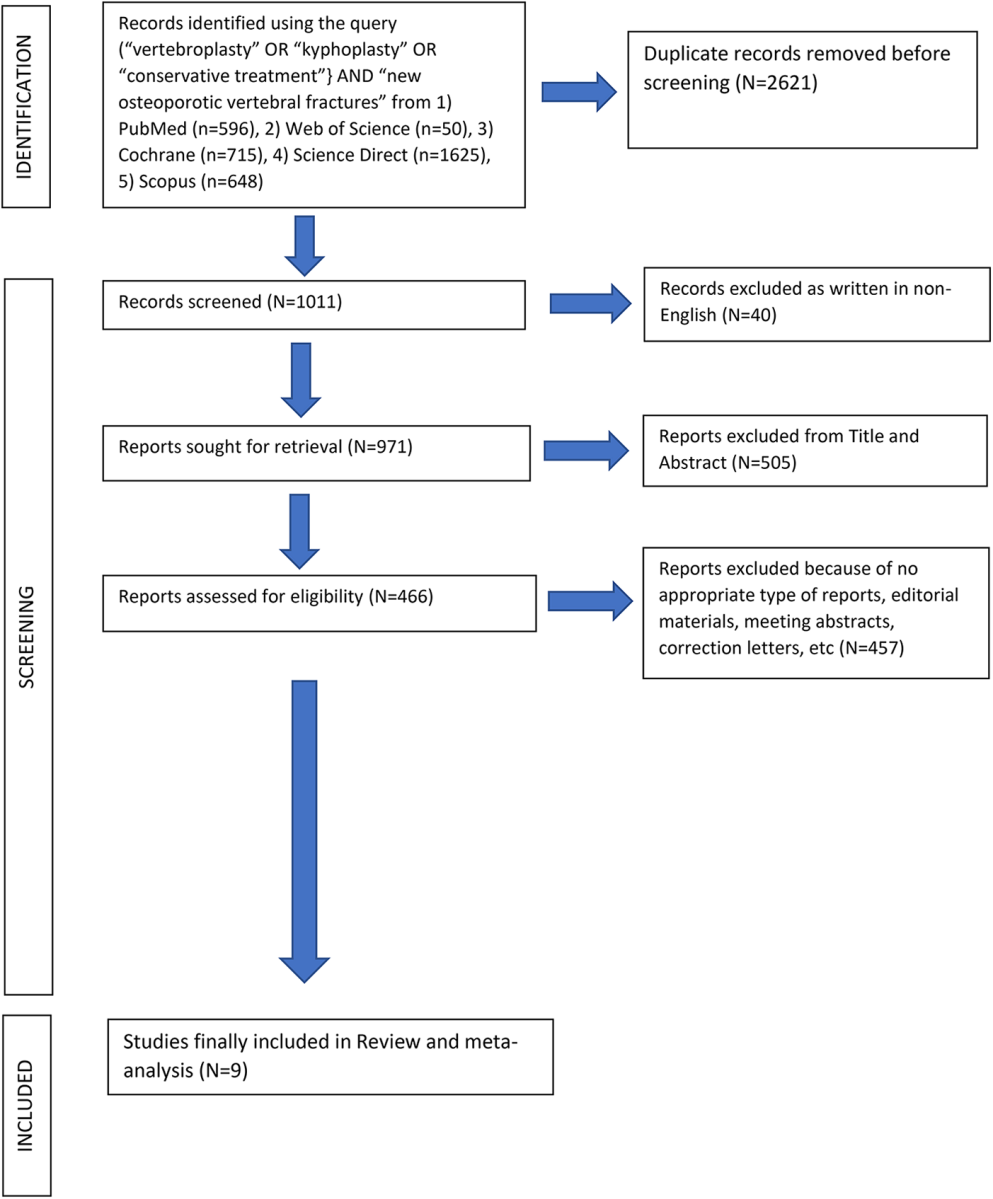
PRISMA flowchart for the systematic review and meta-analysis.

After the computerized search was performed, 3,632 articles were identified from 5 different data bases (PubMed, Web of Science, Cochrane, Science Direct and Scopus). 2,621 papers were identified as duplicates (presented more than once in the search results) and were rejected before screening. In the level 1 screening, 40 records were excluded as these were not written in English language. Of the remaining 971 articles, 505 were excluded reviewing the title and abstract in the screening level 2. From the remaining 466 reports which were assessed for eligibility, 457 articles were excluded in the level III by the unbiased reviewers because of not appropriate type of reports such as editorial materials, meeting abstracts, correction letters, etc. Finally, 9 papers fulfilled all the inclusion criteria and were selected for data extraction and analysis, Figure 1. The 9 studies included in this review were published in the period between 2009 and 2021. Cochrane Collaboration's tool showed low “Risk of Bias” (Table 2) and Newcastle‒Ottawa scale (NOS) showed high quality scores for the retrospective studies (Table 3).

### Studies common characteristics

Countries & centers per study: The studies included in our meta-analysis took place in one or in combinations of totally 8 countries (Iran, Korea, PR China, USA, Switzerland, Netherlands, Belgium, Denmark). Seven studies were single-center studies (5 RCTs, 2 Retrospectives); Patients: There were 1,930 patients in the selected studies. 968 patients received conservative and 962 patients received surgical treatment (PVP or PKP). Age of patients: The reported patients' average age ranged from 64 to 80 years in the surgical group and 63.9–80 years in the CT group; Total OVCFs in both groups: The total number of OVCFs in both groups was 2,154, recorded across 1,930 patients. The ratio of index patients to fractures was 1:1.12; Number of primary OVCFs which were augmented (PVP or PKP): 1,107 vertebrae; Unilateral vs. Bilateral approach for vertebral body augmentation: In the studies reviewed, vertebral body augmentation was performed using different approaches: (a) Unilateral Approach used in 4 studies, (b) Bilateral Approach used in 3 studies and (c) Varied Approach (uni- plus bi-lateral) used in 2 studies; Bone cement (PMMA): The amount of injected PMMA per augmented vertebra ranged from 1 to 9 ml and was reported in only three studies (39, 43, 61). PMMA leakage into the adjacent intervertebral disc: Intradiscal PMMA leakage was reported in 3 out of the 9 studies (39, 43, 62). The reported rate of cement leakage ranged from 0.9% to 33.8%. Adjacent new fractures (ANVFs) after surgical and conservative treatment: All 9 studies reported on the incidence of ANVFs; In the CT group, the ANVFs incidence ranged from 7.8% to 35%, whereas in the surgical (PVP/PKP) group, it ranged from 2.6% to 20.4%. Time lapsed from index fracture and ANVFs occurrence in both groups (Surgical, CT): The average time elapsed from the index fracture to the occurrence of new vertebral fractures (ANVFs) in both the surgical and conservative treatment (CT) groups ranged from 3 to 78 months. Conservative treatment: 7/9 studies describe the non-surgical treatment mode (Table 1).

### Meta-analysis

**Figure 2:**
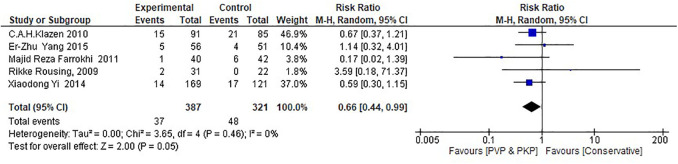
Forest plot for the RCT group. The results indicate a statistically significant difference (*P* < 0.05) between the experimental (PVP/PKP) surgical group and the control (CT) group.

**Figure 3:**
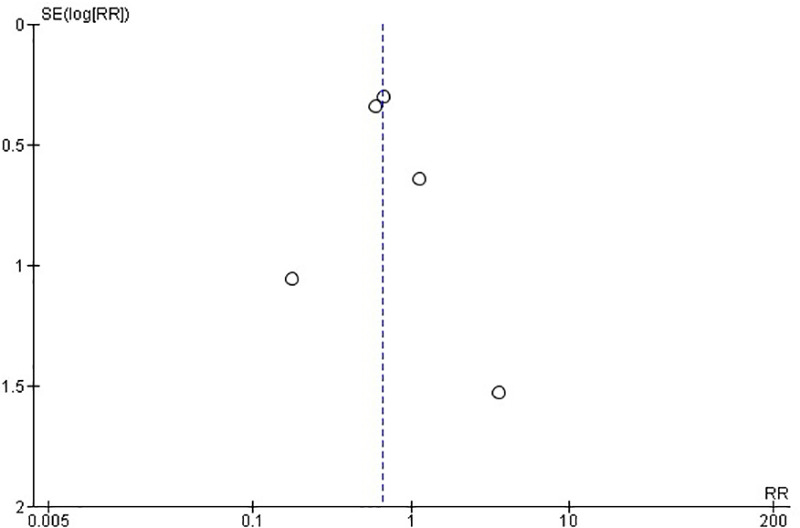
Funnel plot for RCTs, indicating no evidence of publication bias. Egger's test was non-significant (*P* = 0.743).

Regarding the RCTs, the analysis suggests a statistically significant difference (*P* = 0.05) between the surgical (PVP & PKP) group and the conservative treatment (CT) group (Figure 2) in the incidence rate of ANVFs. Specifically, it was expected that patients in the surgical group would have a lower rate of adjacent new vertebral fractures compared to those in the CT group, with a relative risk (RR) = 0.66 (95% C.I: 0.44–0.99; *P* = 0.05). The pooled estimate was calculated using a random effects model with the inverse variance method. The *I*^2^ heterogeneity index was 0%, indicating no significant heterogeneity. Sensitivity analysis for this group showed that the inference could be influenced if certain studies were omitted. The funnel plot (Figure 3) is indicative of the symmetry observed indicating absence of publication bias and the Egger's test was non-significant (*P* = 0.743).

**Figure 4:**
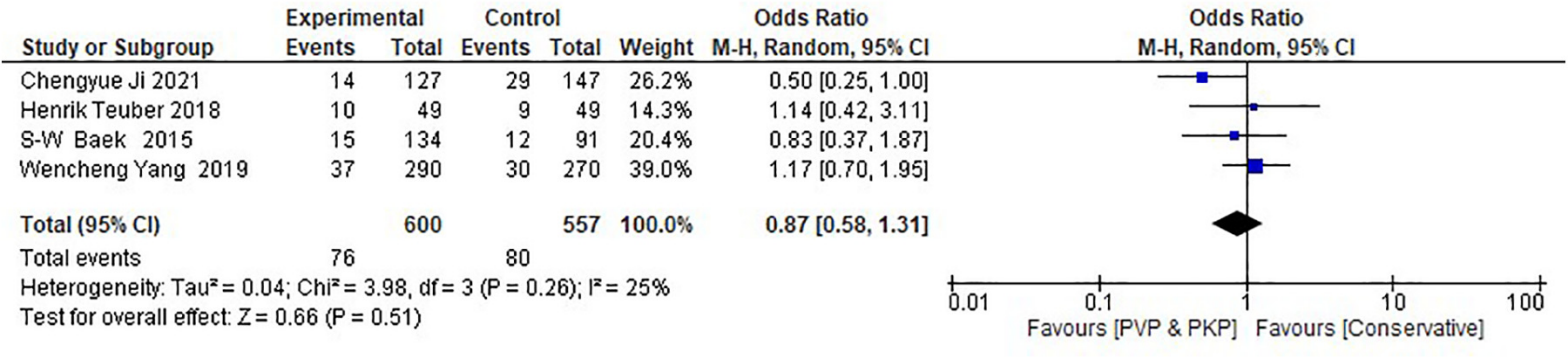
Forest plot for the retrospective studies. The analysis shows no statistically significant difference between the experimental (PVP/PKP) surgical group and the control (CT) group.

**Figure 5:**
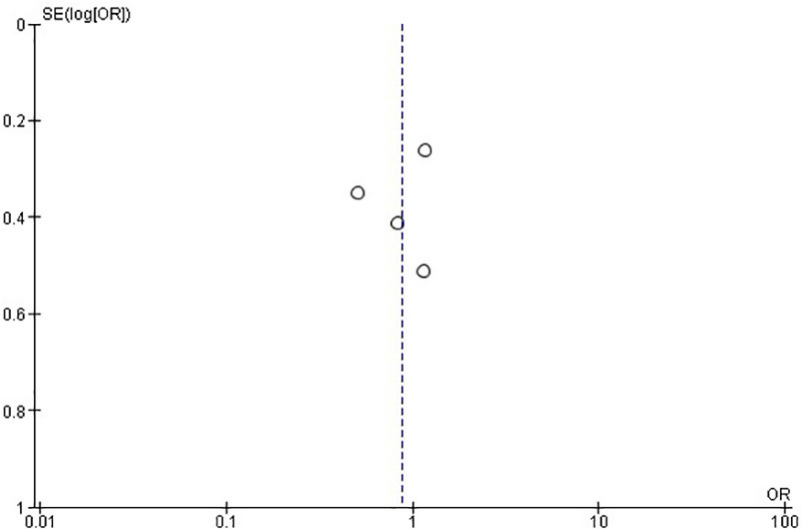
Funnel plot for retrospective studies, suggesting no evidence of publication bias. Egger's test was non-significant (*P* = 0.749).

Regarding the retrospective studies, the analysis indicates no statistically significant differences between the surgical and the CT group in the incidence rate of ANVFs (Figure 4). It was expected that patients in the surgical group would have a similar rate of ANVFs compared to those in the CT group, with an odds ratio (OR) = 0.87 (95% C.I.: 0.58–1.31; *P* = 0.51). The pooled estimate was derived using a random effects model with the inverse variance method. The *I*^2^ heterogeneity index was 25%, which was not statistically significant. Sensitivity analysis for this group showed that the inference remained unchanged, regardless of which studies could potentially be omitted. In the retrospective studies, the funnel plot is indicative of the symmetry observed indicating almost an absence of publication bias and the Egger's test was non-significant with a *P*-value equal to 0.749 (Figure 5).

Differences between the studies, included in this meta-analysis were observed for other parameters as well (Tables 1A,B). Specifically, regarding age means ranged from 64 to 80 years across studies but in all cases the average estimated between the surgical and CTs were close to a mean difference that did not exceed the 2 years, in any study. The total number of participants was different across studies, but always balanced between the two different treatments, while this, relatively small fluctuation, was accounted for through the weights attributed to each study in the synthesis of the results.

**Figure 6:**
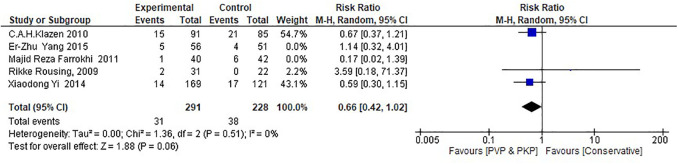
Forest plot for the RCT subgroup analysis examining the correlation between pedicular surgical approaches (unilateral vs. bilateral) in PVP/PKP and the incidence of ANVFs. The unilateral approach has a tendency towards an increased incidence rate of ANVFs; however, this increase is not statistically significant (*P* = 0.06).

The percutaneous unilateral vs. bilateral approach appears to affect the inference, but still the small number of studies included in this analysis does not allow a clearer view than the one stated in the section regarding RCTs (Figure 6). Similarly, subgroups (Tables 1A,B) that could theoretically differentiate the ANVFs outcomes relating to PMMA (amount, cement leakage, or follow up time), cannot be statistically examined due to the small number of studies included in this meta-analysis. It has to be mentioned though that the evidence provided by the authors in this context includes no indications for major differences.

## Discussion

Traditionally, the primary treatment for OVCFs is conservative however this treatment is often associated with a poor quality of life, persistent pain, and complications arising from reduced patient mobility (33, 37, 47–53, 63–68).

A frequent complication of OVCFs, either treated surgically or with CT, is the occurrence of new OVCFs in adjacent vertebra (ANVFs). ANVFs, when left untreated, may decrease furthermore the quality of life and increase the morbidity and mortality in elderly patients (63).

Despite the fact that the majority of OVCFs heal without surgery, a relative recent review reported that 15%–35% of patients with an OVCF suffer from persistent intractable back pain, while severely collapsed OVCFs that may cause neurologic deficit, local kyphosis, or chronic pseudarthrosis frequently require surgery (54). Both CT and percutaneous augmentation methods (PKP, PVP) have advantages and disadvantages, and while there are a variety of trials describing the outcomes and complications of each treatment, there is still debate regarding the incidence rates of ANVFs following operative treatment and CT for OVCFs (69).

The reported major predictive risk factors in OVCFs are vertebral collapse, pseudarthrosis, local kyphotic deformity, and neurologic impairment. If prognosis can be predicted at the early fracture stage, some authors recommend more aggressive treatment options, rather than CT (70).

There is still debate and controversy about the effectiveness of PVP and PKP in comparison to CT regarding the incidence of ANVFs. There are numerous reports on indications, results and complications after PVP or PKP. Yet despite the positive findings seen in these reports, there is still conflicting results about surgical indications for PVP and PKP in treating OVCFs, except for cases that had failed CT (37, 71). In contrast to CT, PVP has been reported to afford rapid relief of back and low back pain, permit ambulation, and improve quality of life, however, there have been few reports concerning the long-term clinical efficacy of PVP (61, 65, 72).

While primary PVP may alleviate back pain, this symptom can occasionally reoccur during follow-up, often due to ANVFs. ANVF remain a topic of debate. Some authors suggest that the augmented vertebra has a different modulus of elasticity or stiffness compared to the adjacent fractured vertebra, resulting in increased forces on the surrounding vertebrae (73). In contrast, others argue that cement interdigitation acts as an internal fixation mechanism, strengthening and restoring the anterior column while reducing the flexion moment on the surrounding vertebral bodies (74).

There is an ongoing debate regarding the incidence of ANVFs after CT compared to PVP/PKP, with RCTs reporting varying outcomes—some indicating lower ANVFs rates after PVP/PKP (23), others showing similar rates (34, 36, 37, 75) and some suggesting a lower ANVFs rate with CT (42, 76).

A previous systematic review found that 17 clinical trials on PVP and 12 clinical trials on PKP reported new vertebral fractures. Of the new vertebral fractures following PVP and PKP, 60% and 66%, respectively, occurred adjacent to the augmented vertebra, though the incidence rates for conservative treatment were not mentioned (70). A meta-analysis by Tian et al. (77) investigated the clinical efficacy of PVP for the treatment of OVCF compared to conservative treatment and found no statistically significant difference in the incidence of adjacent vertebral fractures between the two groups. However, inconsistencies in follow-up durations across studies made direct comparisons challenging. In our systematic review, the incidence of adjacent new vertebral fractures in the conservative treatment group ranged from 7.8% to 35% across 9 clinical trials, while in the surgical group (PVP/PKP), it ranged from 2.6% to 20.4%. The Meta-analysis showed that in the RCTs the new fracture incidence was lower in the patients who received PVP/PKP.

Our study aligns with previous research regarding the wide range of follow-up (70). A retrospective study suggested that ANVFs tend to occur earlier than other new fractures in the rest of the spine (78), while long-term studies comparing CT and PVP yielded conflicting results (42–44).

The primary challenge in conducting a meta-analysis comparing studies on PKP and PVP vs. CT in patients with OVCFs is the lack of standardization in CT treatment options (Table 4). To the best of the authors' knowledge, the existing literature includes only six meta-analyses published between 2013 and 2021, along with one narrative review in 2023, that have compared surgical interventions (PKP, PVP) with CT regarding ANVFs incidence (35, 80–85). All of these studies (35, 80–85) concluded that there is no significant difference in the ANVFs incidence rate between PVP/PKP and CT. Our meta-analysis disclosed no significant difference in ANVFs incidence rates between surgical (PVP/PKP) and CT groups in the retrospective studies, this being in accordance to previous meta-analyses. In contrary, in our meta-analysis, when analyzing the RCTs, the vertebral augmentation (PVP/PKP) showed a significantly (*P* < 0.05) lower ANVFs incidence rate than the CT group. This superiority of interventional treatment (lower incidence rate of ANVFs) in the RCTs group in our meta-analysis, should be evaluated in terms of its significance alongside the benefits highlighted in related research concerning Quality of Life outcomes. This however was not the scope of our meta-analysis and presents a limitation of our study (3, 11, 52, 79, 80, 84–87).

**Table 4:** Critical data from studies referred in the discussion section.

Authors	Year of publication	Type of studies included	Country	Patient groups compared	Type of conservative treatment (CT)	Outcomes	Follow-up period	Results	New adjacent vertebral fractures
A. J. Láinez Ramos-Bossini (87)	2021	RCTs	Spain	PVP vs. CT & placebo	Not specified	Pain relief, improvement in functional disability, and quality of life	>6 months	PVP showed significant benefits over CT for all outcomes, but only minor differences compared to placebo	Not reported
Sascha Halvachizadeh (83)	2021	RCTs	Switzerland	PVP, PKP vs. CT	Not specified	Pain reduction, risk of adjacent-level fractures, and quality of life (QOL)	Short-term (weeks), mid-term (months), long-term (>1 year)	PVP and PKP provided better pain relief but did not increase the risk of adjacent-level fractures or impact QOL	No increased risk of adjacent-level fractures after any treatment
Jintao Liu (52)	2013	RCTs	China	PVP vs. CT (including optimum pain treatment, optimal medical therapy and conservative treatment)	Not specified	Pain reduction comparison between PVP and CT	12 months	PVP significantly reduced pain compared to non-operative therapy at all times	Not mentioned
Lin Zhang (81)	2019	RCTs	China	PVP vs. CT	Bed rest, brace, anti-osteoporotic medication, analgesics, physiotherapy, conservative pain treatment, simulated procedure without PMMA	Effectiveness of PVP and CT in pain management and functional outcomes for OVCF patients	1–36 months	Patients treated with vertebroplasty experienced better pain relief and quality of life without increasing the incidence of new fractures compared to CT	No significant difference in new fractures between PVP and CT
Lin Xie (35)	2017	RCTs	China	PVP vs. CT	Bed rest, brace, anti-osteoporotic medication, analgesics, physiotherapy, conservative pain treatment, simulated procedure without PMMA	Pain relief (1 week to 6 months), quality of life (RDQ, ED-5Q, and QUALEFFO), and rate of adjacent vertebral fractures	3–6 months	PVP provided greater pain relief than CT in the early period, but no significant difference in RDQ and ED-5Q scores	No statistical difference in the rate of adjacent vertebral fractures between PVP and CT
Ryan Mattie (11)	2016	RCTs	USA & Finland	PVP vs. CT	Analgesics, calcitonin, intrathecal fentanyl, brace treatment, physiotherapy, osteoporotic medication	Pain levels at different time intervals	1–36 months	PVP provided significantly lower pain levels compared to CT for up to 1 year postoperatively	Not mentioned
Paul A. Anderson (84)	2013	RCTs	USA	PVP vs. CT	Optimal pain management optimal medical treatment, non-surgical care, sham, needle insertion, local anesthetic injection adjacent to the vertebral body	Pain relief, functional recovery, and quality of life	6–12 months	Cement augmentation showed superior pain relief, functional recovery, and quality of life compared to non-operative or sham treatment	Conflicting results: 3 studies favored CT (fewer fractures), 3 studies favored PVP (no statistical significance)
S. Lou (85)	2019	RCTs	China	PVP vs. CT	Not described	Pain relief at different time intervals and rate of new fractures	1–12 months	Pain scores were similar between the PVP and sham injection groups, but the effect size of PVP increased over time	No difference in the risk of new vertebral fractures between PVP and CT
Wei-Hsin Yuan (86)	2016	RCTs	Taiwan, ROC	PVP, PKP vs. CT	Not described	Comparison of vertebroplasty and kyphoplasty outcomes with conservative treatment	2 weeks to 36 months	PVP & PKP improved functional outcomes significantly more than CT; BKP had a greater effect on quality of life than CT, but no difference was found between PVP and CT	Not mentioned
Zuo et al. (3)	2018	Meta-analysis	China	PVP, PKP, nerve block (NB) vs. CT	Not described	Comparison of vertebroplasty and kyphoplasty outcomes with conservative treatment	Short-term (∼4 weeks), long-term (∼12 months)	PVP showed superior pain relief compared to CT for acute/subacute OVCFs	Not mentioned
Sanli et al. (79)	2020	Systematic review & Meta-analysis	Netherlands	PVP vs. CT or sham procedures	Not described	Pain, disability, and quality of life (QOL)	6 months	Significant improvements in PVP over CT	Not mentioned
Zhu et al. (80)	2019	Meta-analysis	China	PVP, PKP vs. CT	Not described	Pain, ODI, and RMDQ scores	2 weeks to 49.4 months	PVP significantly reduced pain, ODI, and RMDQ compared to CT. PKP and PVP had similar efficacy for pain relief and functional improvement	No significant differences in subsequent vertebral fractures across treatment groups; PKP had the highest probability (34.75%) of reducing fracture risk

Furthermore, in our meta-analysis, certain parameters varied from study to study. More specifically, the average age ranged from 64 to 80 years across the studies, but in all cases, the age differences between the surgical and CT groups were minimal, with a mean difference of no more than 2 years in any study. The total number of participants varied across the nine studies but remained balanced between the interventional and CT groups. This relatively small variation was accounted for the weights assigned to each study in the result synthesis. In our meta-analysis, the choice between a unilateral or bilateral pedicular approach for vertebral body augmentation appears to marginally influence the incidence of new fractures (*P* < 0.06), in favor of unilateral approach. This could be because of the two distinct augmentation techniques (PKP, PVP) used in the studies included in our meta-analysis. However, due to the limited number of studies, a more detailed understanding beyond what is discussed in the RCT section is not feasible. Similarly, potential subgroups that could theoretically affect new fractures generation—such as PMMA cement volume per vertebra, cement leakage, or follow-up duration—could not be statistically analyzed because of the limited number of included studies. Nevertheless, the evidence presented does not suggest any major differences in this regard. Conducting a meta-regression analysis for multiple subgroups creation, based on various potential risk factors for ANVFs would require separate analyses for RCTs and retrospective studies. As Thompson and Higgins (88) noted, a meta-regression should generally not be performed when fewer than ten studies are included in a meta-analysis. Since our meta-analysis included only nine studies, RCTs and retrospective studies were analyzed separately due to their distinct data collection methods.

Selected meta-analyses including RCTs exclusively have compared surgical treatments (PVP, PKP) with CT in terms of functional outcomes after treating of OVCFs and reported significantly better functional outcomes and pain relief in the first postoperative year following PVP/PKP (3, 11, 52, 79, 80, 84–87). In contrary to our results in the RCTs group, all these meta-analyses showed no significant differences in the incidence of ANVFs between the surgical and CT groups (Table 4). This difference could be due to the low number of RCTs included in our meta-analysis.

Some authors have emphasized the lack of standardized management strategies for OVCFs and recommended improving the quality of guidelines through multimodal approaches, including CT, surgery, and osteoporosis treatments, such as medications that promote fracture healing (54). However, numerous reports have demonstrated the beneficial effects of PVP without increasing the risk of ANVFs associated with this procedure when compared to CT in treating OVCFs (89). In our meta-analysis, the analysis of the RCTs suggests a statistically significant (*P* < 0.05) difference in the rate of ANVFs in favor of surgical group, however this was not shown in the retrospective studies group too. According to previous studies, the use of PMMA in PVP effectively stabilizes the fractured vertebral body, leading to pain relief and to an improved ability of performing daily activities (61, 72).

Whether PKP or PVP are associated with lower rates of ANVFs is a widely debated issue. A meta-analysis comparing PKP and PVP concluded that the occurrence of ANVFs in the PKP group did not differ from the PVP group (90).

There are still controversies in the literature regarding factors that may affect the outcomes following PVP and PKP. The time from OVCF to treatment appears to be an important factor that likely influences the outcome. Studies with shorter durations between the onset of pain and randomization tended to show greater effects favoring PVP. Similarly, the diagnostic criteria for enrollment varied, and studies using MRI edema as a criterion showed larger effect sizes in favor of PVP. Despite including studies with these two less favorable inclusion criteria, the pooled results remained significant (84).

Regarding the pros- and cons- of the unilateral compared to the bilateral approach for percutaneous augmentation, both biomechanical data (91–93) and clinical series (94–97) suggest that the unilateral procedure is safe and effective compared to bilateral augmentation. Additionally, comparative studies claim no significant difference in clinical or radiological parameters between uni- and bilateral augmentation (98–100). In our meta-analysis, the RCT subgroup analysis explored the correlation between pedicular approaches (unilateral vs. bilateral) in PVP/PKP and the incidence of new fractures. The unilateral approach appears to decrease the likelihood of new fractures; however, this decrease is only marginally significant (*P* = 0.06).

The volume of cement to be injected for optimal results remains a point of debate among different authors. Biomechanical studies suggest that smaller cement volumes may be sufficient to restore stiffness to pre-existing damaged levels (101), while others recommend larger volumes to restore vertebral strength and stiffness (102, 103). Some authors have proposed that smaller amounts of cement may be enough to resolve clinical symptoms (104). However, growing evidence suggests that larger cement volumes are associated with better pain resolution (105) and improved restoration of sagittal alignment (106, 107). In the studies included in our meta-analysis, the amount of PMMA injected per vertebra varied significantly among studies, and unfortunately it was reported in only three studies (39, 43, 61) regarding ANVFs.

## Conclusion

This meta-analysis for the selected RCTs shows that vertebral augmentation is associated with lower incidence ANVFs compared to CT. On the other hand, in the retrospective studies group there was no significant difference in the incidence of ANVFs between the two treatment groups (CT vs. PKP/PVP). Variations in study parameters, such as patient demographics and surgical techniques, may have affected these results. Further high-quality studies are needed to better understand the long-term effects of different treatment strategies on the incidence of ANVFs.

Future research should adopt standardized diagnostic criteria (e.g., roentgenograms, MRIs, clinical evaluation) to ensure comparable fracture-to-treatment timelines. Additionally, longer follow-up periods are needed to better assess ANVFs rates. Standardizing factors such as PMMA volume, cement injection techniques for PVP/PKP, and conservative treatment protocols will help to reduce variability.

### Limitations

This systematic review and meta-analysis included both retrospective studies and RCTs. Both groups consisted of selected comparative studies with similar populations in the experimental and control groups. Cochrane Collaboration's tool showed low “Risk of Bias” for RCTs and Newcastle‒Ottawa scale showed high quality scores for the retrospective studies. The included studies employed various conservative treatment (CT) methods and follow-up protocols. The time from the initial fracture to the development of ANVFs varied across studies in both treatment groups. Two commonly used vertebral body augmentation techniques—PVP and PKP—were applied, sometimes within the same study, despite their distinct surgical effects on fractured vertebrae and associated complications (e.g., kyphosis reduction, cement leakage). The reported surgical approach (unilateral vs. bilateral pedicular access) and the amount of PMMA injected per augmented vertebra also varied between studies. A significant limitation of this review is the absence of functional outcome measures (e.g., pain, quality of life, ODI) in all nine included studies. Since the primary concern for elderly patients is post-treatment quality of life, the lack of such data limits the clinical relevance of the findings. Despite these limitations, our meta-analysis has several strengths. It includes studies from six different countries, demonstrates low publication bias in both retrospective and RCT studies, and involves a comparable number of patients undergoing CT or surgical treatment—enhancing the representativeness of the results. Additionally, the high quality of the included studies supports the validity of our conclusions. These limitations do not compromise the reliability of this meta-analysis.

## Data Availability

The datasets presented in this study can be found in online repositories. The names of the repository/repositories and accession number(s) can be found in the article/Supplementary Material.
